# Resting state connectivity in people living with HIV before and after stopping heavy drinking

**DOI:** 10.3389/fpsyt.2023.1102368

**Published:** 2023-05-17

**Authors:** Joseph M. Gullett, Jason DeFelice, Veronica L. Richards, Eric C. Porges, Ronald A. Cohen, Varan Govind, Teddy Salan, Yan Wang, Zhi Zhou, Robert L. Cook

**Affiliations:** ^1^Center for Cognitive Aging and Memory, Department of Clinical and Health Psychology, University of Florida, Gainesville, FL, United States; ^2^Department of Epidemiology, University of Florida, Gainesville, FL, United States; ^3^Edna Bennett Pierce Prevention Research Center, The Pennsylvania State University, University Park, PA, United States; ^4^Department of Radiology, University of Miami Miller School of Medicine, Miami, FL, United States; ^5^University of Miami, Coral Gables, United States

**Keywords:** human immunodeficiency virus, alcohol use, resting-state functional magnetic resonance imaging, contingency management, cessation

## Abstract

**Background:**

Heavy alcohol use in people living with HIV (PLWH) has widespread negative effects on neural functioning. It remains unclear whether experimentally-induced reduction in alcohol use could reverse these effects. We sought to determine the effects of 30-days drinking cessation/reduction on resting state functional connectivity in people with and without HIV.

**Methods:**

Thirty-five participants (48.6% PLWH) demonstrating heavy alcohol use attempted to stop drinking for 30 days via contingency management (CM). MRI was acquired at baseline and after thirty days, and functional connectivity across five resting-state fMRI (rsfMRI) networks was calculated with the Conn toolbox for Matlab and examined in relation to transdermal alcohol concentration (TAC) recorded by the ankle-worn secure continuous remote alcohol monitor (SCRAM) and self-reported alcohol use (timeline follow-back; TLFB). Associations between alcohol use and reduction, HIV status, functional connectivity, and change in functional connectivity across five major rsfMRI networks were determined relative to the pre- and post-CM timepoints.

**Results:**

Baseline resting-state functional connectivity was not significantly associated with average TAC-AUC during the pre-CM period, though higher self-reported alcohol use over the preceding 30 days was significantly associated with higher baseline connectivity within the Dorsal Attention Network (DAN; p-FDR < 0.05). Baseline connectivity within the Salience network was significantly negatively related to objective drinking reduction after intervention (DAN; p-FDR < 0.05), whereas baseline connectivity within the Limbic network was positively associated with self-reported drinking reduction (p-FDR < 0.05). Change in between-networks functional connectivity after intervention was significantly positively associated with biosensor-confirmed drinking reduction such that higher reduction was associated with stronger connectivity between the limbic and fronto-parietal control networks (p-FDR < 0.05). PLWH with lower DAN connectivity at baseline demonstrated poorer alcohol reduction than those with higher DAN connectivity at baseline.

**Discussion:**

Lower resting-state functional connectivity of the Salience network significantly predicted stronger drinking reduction across all participants, suggesting a potential biomarker for reduced susceptibility to the environmental and social cues that often make alcohol use reduction attempts unsuccessful. Increased between-networks connectivity was observed in participants with higher alcohol reduction after CM, suggesting a positive benefit to brain connectivity associated with reduced drinking. PLWH with lower baseline DAN connectivity may not benefit as greatly from CM for alcohol reduction.

## Background

People living with human immunodeficiency virus (PLWH) demonstrate a 29.8% point-prevalence of alcohol use disorder (AUD) (1), which is considerably high given the implications heavy alcohol use has on the immune system (2) and on the metabolism of antiretroviral drugs (3) in this population. Not only has heavy alcohol use been associated with increased risk of all-cause mortality among PLWH compared to those without HIV (4), its impact is widespread across domains of health, social, cognitive, and brain functioning (5–11). In fact, heavy alcohol use has been shown to have more deleterious effects on cognitive and neural domains in PLWH when compared to people without HIV (12). Specifically of interest is the effect of these comorbid conditions on neural functioning, where our group has demonstrated a number of effects of heavy alcohol use in HIV including reduced frontal white matter integrity (13) and reduced functional connectivity of frontal and parietal attentional networks (14). Despite the risk factors associated with heavy drinking, studies of drinking cessation and its effects on brain function in PLWH are quite limited, thus necessitating further work to highlight the clinical relevance of functional connectivity changes in drinking cessation among people living with and without HIV.

### Introduction to neural networks under study

Neural networks in the brain are large-scale functional and structural networks composed of groups of interconnected neurons and brain regions that work together to process and integrate information. Some of the most commonly examined resting-state networks in the context of alcohol use and HIV include the default mode network (DMN), the dorsal attention network (DAN), the cingulo-opercular network (CON/Salience network), the limbic network, and the fronto-parietal control network (FPCN). The DMN comprises regions including the medial prefrontal cortex, posterior cingulate cortex, and the inferior parietal lobule and is active when an individual is at rest, suggesting involvement in intrinsic activity. The DMN is also thought to play a role in regulating attention and monitoring the external environment for salient information (15). The DAN includes several regions of the parietal and frontal cortices, including the superior parietal lobule, intraparietal sulcus, and frontal eye fields, and has been implicated in controlling attention and selecting relevant information from sensory input. It is primarily responsible for directing attention to visual and spatial information, and for the control of voluntary eye movements (16). The cingulo-opercular (e.g., salience) network is a neural network in the brain that is involved in detecting and filtering important or salient sensory and cognitive information from the environment or internal mental states. It includes the anterior insula, dorsal anterior cingulate cortex (dACC), and the fronto-insular cortex (17). The limbic network is a group of interconnected brain structures (e.g., the hippocampus, amygdala, hypothalamus, thalamus, and cingulate gyrus) that play a key role in emotional processing, motivation, learning, and memory (18). The FPCN is involved in cognitive control, attentional processing, working memory, planning, goal-directed behavior, and decision-making, and consists of regions in the prefrontal and parietal cortices that are interconnected by white matter tracts, enabling the rapid transmission of information between these regions (19). There is an abundance of evidence suggesting alterations in the resting-state functional connectivity (rsFC) of these neural circuits associated with AUD and HIV.

### Functional connectivity in AUD

AUD is thought to be caused by a compulsive “drive” toward alcohol consumption (20) as well as an inability to inhibit alcohol consumption, which correspond with increased activity in the appetitive drive networks (a subset of the limbic network associated with reward processing) and decreased activity in brain regions which mediate executive control compared to individuals without AUD (21–23). Resting-state fMRI research in alcohol use and disorders consistently implicates the DMN, the FPCN (also referred to as the Central Executive Network), and the salience network (20). For example, one group found that alcohol use disorder was associated with increased connectivity between fronto-parietal regions involved in cognitive control and decreased connectivity between nine regions of the Salience network involved in reward processing and emotional regulation (Kamarajan, 2022). Similarly, increased between-network rsFC among the executive control network, Salience network, and Limbic regions including the striatum and amygdala as well as increased within-network connectivity in the Salience network, DMN, executive control network, and Limbic network has been found in individuals with AUD (Le Berre et al. 2017). Additionally, rsfMRI studies have shown decreased synchronicity in the posterior cingulate and cerebellar regions (i.e., circuitry of the DMN and DAN) in people with AUD, indicating compromised functional connectivity (28). These rsFC differences may contribute to the cognitive and emotional deficits commonly observed in individuals with chronic alcohol use. However, these alterations in activation may be reversible after prolonged reduction in alcohol use.

### Connectivity changes after drinking reduction

Following drinking reduction and abstinence, individuals with AUD experience altered neural circuits of stress and reward modulation (e.g., increased limbic network circuitry), making them highly sensitive to stress, anxiety, low mood, autonomic nervous system disruption, fatigue, and sleep problems (29, 30). Functional MRI (fMRI) studies have shown that participants with AUD who stop drinking demonstrate greater activity in limbic-striatal regions (e.g., the limbic network) associated with emotional processing and lower activity in the medial frontal and cingulate regions, particularly in the caudate and posterior cingulate regions associated with emotional regulation, self-control, and executive functioning (31–33). These findings either suggest negative implications of drinking reduction in this population, specifically increased emotional reactivity and decreased self-monitoring, which may make it more difficult to maintain abstinence, or indicate a lack of reversibility of functional connectivity changes associated with AUD. Interestingly, individuals with alcohol use disorders who successfully completed detoxification showed higher connectivity of the DMN, the FPCN, and the salience network compared to those who dropped out of treatment (34). However, several studies have identified evidence for compensatory mechanisms in the resting state networks of long-term abstinent individuals with AUD. One study found increased synchrony in the inhibitory control network (i.e., a subset of the FPCN) and reduced synchrony in the appetitive drive reward network in long-term abstinent persons with AUD when compared to controls without AUD (J. Camchong, Stenger, and Fein 2013), suggesting alterations in connectivity with prolonged drinking reduction. Similarly, Chanraud et al (2011) found resting state synchronicity in posterior cingulate and cerebellar regions (i.e., circuitry of the DMN and DAN) in abstinent persons with AUD compared to age-matched healthy controls who did not meet criteria for AUD connectivity that improved with longer durations of abstinence (Chanraud et al. 2011). Similarly, another study found increased synchrony in the inhibitory control network (i.e., a subset of the FPCN) and reduced synchrony in the appetitive drive reward network in long-term abstinent persons with AUD when compared to controls without AUD (35), suggesting alterations in connectivity with prolonged drinking reduction. While there is evidence for neurobiological changes following drinking reduction and abstinence in adults with AUD, the length of abstinence needed to achieve these changes is unknown. Similarly, there is a dearth of literature investigating these effects in PLWH.

### Connectivity in PLWH

fMRI techniques have been widely used to investigate the neural basis of HIV-associated neurocognitive disorders. In fact, previous fMRI studies have demonstrated marked functional connectivity differences in PLWH, independent of alcohol consumption. For example, a recent meta-analysis found consistent alterations in the fronto-striatal-parietal sub-networks (functionally related to the FPCN), including hyperactivation in the left inferior frontal gyrus and caudate nucleus, which are associated with cognitive impairment, disease progression, and treatment outcomes (36). Further, reduced within-network connectivity of the DMN, the FPCN, and the salience network is implicated in PLWH, in addition to inter-network differences in the DAN and the salience network (37). In studies utilizing diffusion tensor imaging (DTI), duration of HIV infection has been independently associated with white matter injury, especially in frontal projections of the corpus callosum and thalamus (38). Importantly, fronto-striatal and fronto-parietal circuits implicated in HIV progression are functionally involved in the inhibition and regulation of appetitive, attention, impulsive, and emotional responses and behaviors (39), and therefore may be altered by substance abuse. Indeed, in a prior rsfMRI study, our group has shown in a prior resting-state fMRI study lower fronto-parietal connectivity (e.g., FPCN) and increased connectivity between attention/working memory networks (i.e., the DAN) and mesolimbic regions (i.e., the limbic network) critical to addiction in PLWH with chronic alcohol abuse (14). Taken together, previous research indicates reduced white matter integrity in regions important for inhibition and regulation of alcohol consumption as well as increased connectivity between regions implicated in appetitive drive toward alcohol consumption, suggesting a compounding of effects in individuals with HIV and alcohol use disorder.

### Alcohol monitoring and interventions

Behavioral interventions focusing on reducing the frequency of harmful alcohol use are becoming increasingly common. Contingency management (CM) is one of the most effective evidence-based treatment approaches directly addressing substance use disorder that encourages drinkers to alter their drinking behavior to reduce the probability of alcohol-related consequences (40, 41). CM is a behavioral treatment based on operant conditioning principles that gives participants rewards in the form of cash, prizes, or vouchers to reinforce positive behaviors, such as alcohol abstinence (42). This operant conditioning approach is often preferred by patients and their clinicians (43, 44), and contingency management as a harm-reduction intervention produces similar health outcomes as those who abstain from alcohol (45, 46).

Technologies that allow continuous monitoring of alcohol use have been developed and combined with the CM approach to objectively monitor drinking reduction, such as transdermal alcohol detection. This method senses the 1% of ingested alcohol that is secreted through the skin via sweat glands and diffusion (47), allowing for the separation of heavy drinking episodes from lower and moderate drinking levels (48, 49). One device, the Secure Continuous Remote Alcohol Monitoring bracelet (SCRAM, Alcohol Monitoring Systems; AMS), which is regularly worn by court-referred offenders, has been established as valid in controlled laboratory and field trials (50, 51). This device contains three sensors that assess contact with the skin, skin temperature, and perspiration once every 30 min. As a noninvasive measurement approach, the SCRAM measures the concentration of alcohol in insensible perspiration, providing estimates of the frequency and quantity of alcohol consumption over extended periods of time within the participant’s natural environment while avoiding the limitations of frequent testing and self-report.

Transdermal alcohol detection devices, such as the SCRAM, have shown a strong correlation (*r* = 0.84) between area under the curve (AUC) values and breath alcohol content (BrAC) (51). The SCRAM-CAM (Secure Continuous Remote Alcohol Monitor Continuous Alcohol Monitoring; Alcohol Monitoring Systems, Inc., Littleton, CO) anklet is the most commonly used and well-validated alcohol biosensor available (52–54). The SCRAM device can reflect blood alcohol concentration (BAC) within a drinking event continuously and in near real-time (55) by plotting the transdermal alcohol content (TAC) curve, as well as the AUC value representing the geometric area beneath the TAC curve (56). Peak TAC represents the highest level of TAC reached during a drinking episode (48), while area under the curve (TAC-AUC) represents an individual’s total exposure to alcohol during a drinking episode (57). Studies report that TAC is significantly associated with self-reported drinking with correlations ranging from 0.30 to 0.74 (58–61). In a laboratory setting, peak TAC and TAC-AUC have strong correlations to breath alcohol concentration (0.6–0.9) (51). However, SCRAM data, much like BrAC, cannot yet be directly equated into number of drinks consumed. Thus, the equivocation of SCRAM data to real-life drinking level in cross-sectional research is limited, and within-subjects, longitudinal data analysis may be more appropriate.

In the present study, we sought to determine: (1) the baseline associations between resting-state functional MRI (rsfMRI) connectivity, self-reported and objective alcohol use, and HIV, (2) changes in rsfMRI connectivity after 30 days of attempted abstinence from heavy alcohol use (including reduction of alcohol use), and (3) how rsfMRI connectivity alterations after 30 days attempted abstinence from heavy alcohol use differ between PLWH and people without HIV. We propose the examination of five resting-state networks (DMN, DAN, CON/Salience, limbic, and FPCN) in the present study examining the effects of alcohol reduction in individuals exhibiting current heavy alcohol use who may or may not carry a diagnosis of HIV. Prior to data analysis, we hypothesized that participants living with HIV will demonstrate lower rsfMRI connectivity within regions comprising frontal networks, including the Fronto-Parietal Control Network (FPCN), Default Mode Network (DMN), and Limbic, but will be equivalent to participants without HIV in remaining resting-state network (CON/Salience). Further, we hypothesized that resting-state connectivity will be negatively associated with self-reported drinking severity at baseline (prior to intervention), and that associations between resting-state connectivity and drinking severity will be enhanced in participants living with HIV compared to participants without HIV. With regard to changes after the intervention, we hypothesized that participants with stronger abstinence as measured by lower self-reported drinking and a lower TAC-AUC over the 30-day period will demonstrate enhanced rsfMRI connectivity across FPCN, DMN, and Limbic networks. We also hypothesized that participant HIV status will serve to enhance connectivity improvements associated with abstinence, such that those with HIV will see larger relative connectivity changes compared to baseline.

## Methods

### Participants

Participants were part of the 30-Day Challenge study, which is a study to test the effects of alcohol reduction through CM in older adults with heavy alcohol use living with or without HIV who are interested in reducing their alcohol intake. All in-person procedures were conducted at either the University of Miami or Florida International University. To be eligible, participants had to be between 45 and 75 years old, living with or without HIV (confirmed via medical record, medication, or blood test); drink heavily (≥14 drinks/week for women, ≥21 drinks/week for men) in the past 30 days; speak English; be willing to participate in CM to reduce their alcohol use; and be willing to wear the SCRAM for at least 30 days. Participants may have been in treatment for alcohol use in the past, but must not have been actively enrolled in another treatment program or research study at the time of their participation. A total of 39 participants completed MRI at both the baseline and 30-day time points. Of these, one subject was removed due to corrupt rsfMRI data, one was removed for rsfMRI global signal change greater than 3SD from the group mean, and two were removed for rsfMRI motion parameters exceeding 3SD of the group mean, resulting in a final sample of 35 participants. Of the 35 participants in the study, 18 were living with HIV and 17 were people without HIV.

### Objective alcohol use monitoring

As described in our group’s previous work (60), after providing consent participants were entered into an enrollment eligibility phase in which they would wear the SCRAM biosensor for a pre-contingency management (pre-CM) “test week” (hereafter referred to as the Pre-CM period) during which they provided self-reported drinking information. The purpose of the pre-CM period was to confirm that the participant did meet heavy drinking criteria, that they could tolerate the SCRAM monitor, and to demonstrate that the monitor accurately detected drinking days prior to participants entering a CM period. The SCRAM monitor was installed on the participant’s preferred leg and locked in placed once the participant was ready to leave the lab. The participant was given instructions about the monitor, including not to submerge the device in water, to avoid using alcohol-based items (e.g., perfume, bug spray), and not to wear socks under the monitor. Participants were instructed to drink as they normally would during the pre-CM period for all but 1 day of their choosing, in which a day of abstinence was required to assure that the monitor could accurately distinguish between drinking days and abstaining days. During the Pre-CM period, a research assistant called the participant at the end of the week and collected information about self-reported drinking, including the number of drinks on each drinking day. When the participants returned at the end of the pre-CM period, the data on the ankle monitor were uploaded to the SCRAM system using the DirectConnect device. As detailed in a recent work by our group, “TAC data were collected by the SCRAM-CAM and transmitted to the AMS server using the company’s DirectConnect device. Data were downloaded from the server through a password-protected portal. The Transdermal Alcohol Sensor Data Macro (TASMAC Software) read and interpreted data from the SCRAM” (62). The participant took the DirectConnect device home so that the research assistant could check their daily data. The daily compensation was calculated based on their daily data using the social day from 6:00 am to 6:00 am.

### Intervention: contingency management

Contingency management (CM) was implemented in the present study after the pre-CM period through direct payment for alcohol abstinence. Abstinence was determined on a daily basis through remote monitoring (SCRAM) with distribution of funds to the participant upon confirmation of abstinence by a team member. After the pre-CM period, the participant completed a baseline assessment that included neuropsychological testing, additional questionnaires, and blood testing. Subsequently, participants began the 30-day enrollment “challenge” period during which continuous monitoring of alcohol use was enacted using the SCRAM device. At the conclusion of the 30-day CM period, participants again completed all measures from the baseline assessment listed above.

Payments: On the first day of sobriety, a participant would receive $5. For each consecutive day after their payout, it would increase by $1 (e.g., on day one, participants receive $5, day two participant receives $6, etc.) at day seven, they can receive a total of $45 plus a bonus of $25 for maintaining abstinence for seven consecutive days. The bonus payments increase by $20 every 7 days. Therefore, at 14 days of abstinence, their bonus would be $40. The max amount paid for maintaining abstinence throughout a challenge (30 days) could be a total of $440 (not including payout for assessment visits), and a minimum payout could be $0 if the participant had a drinking episode every day. As for the payouts, the participants often chose to receive payments every 8 days (after earning a bonus) to take advantage of the lump sum of money earned for maintaining abstinence. During enrollment, participants were asked about their preferred method of funds transfer and frequency of payments to collect for adjustments and bonuses.

### Analysis of subjective alcohol use

The timeline follow-back (TLFB) is a self-report measure of alcohol consumption over the past 30 days which is typically used to calculate the average number of drinks per day over the prior month (63). The process involves asking participants to recount their drinking behaviors in terms of number of drinks as a team member moves across a calendar, referencing anchors and reminders (e.g., holidays) about what a participant did each day to assist with memory. For the purposes of this study, 30-day timeline follow-back was completed with each participant at the baseline timepoint and the 30-day follow-up timepoint. The average number of drinks per day was then calculated by dividing their total reported number of drinks by a constant of 30. This provided a TLFB-30 data point at the baseline visit as well as at the 30-day follow-up visit. Lastly, a percent reduction value was calculated from these data such that their post-intervention 30-day TLFB total was subtracted from the pre-intervention 30-day TLFB total and divided by the pre-intervention total.

$$\frac{PreInterventionTLFB-PostInterventionTLFB}{PreInterventionTLFB}$$

### Analysis of objective alcohol use monitoring

The Transdermal Alcohol Sensor Data Macro (TASMAC Software) (64) was utilized to read and interpret data from the SCRAM biosensor. The TASMAC integrates previously published criteria designed to be more sensitive than the default SCRAM criteria (established by the AMS Inc.) to detect drinking episodes (65). The present study used TAC-AUC generated by the TASMAC as the primary estimate of average daily alcohol use over the test week and 30-day test period. Specifically, daily TAC-AUC was calculated as the sum of all TAC-AUC from detected drinking episodes that began on the SCRAM-detected drinking day. If there were multiple drinking episodes within a single drinking day, TAC-AUCs were summed. For the purposes of analysis, three variables were computed, including (1) the TAC-AUC average over the seven drinking days prior to the intervention, (2) TAC-AUC average over the final 7 days of the 30-day intervention period, and (3) the percent reduction from pre- to post-intervention calculated through the formula:

$$\frac{PreIntervention\;TAC\_AUC-PostIntervention\;TAC\_AUC}{PreIntervention\;TAC\_AUC}$$

The rationale for the use of the TAC-AUC during the final 7 days of the 30-day intervention is that doing so creates a time point most proximal to the acquisition of neuroimaging and equivocal both in duration and proximity to the neuroimaging as the pre-CM TAC-AUC data.

*Specific abstinence calculation procedure*: To determine whether the participant remained abstinent based on objective SCRAM data, we used sensitive criteria originally devised by Barnett et al., as follows:

1. The peak of TAC data must reach = > 0.02 at least once (basic criteria).
  and
2. Absorption rate must be less than 0.05.
  or
3. For a peak <=0.15, elimination rate must be (>0.003 and < 0.025).

For a peak >0.15, elimination rate must be (>0.003 and < 0.035).

A drinking episode must meet rule 1 and 2 (based on absorption rate) OR rule 1 and 3 (based on elimination rate). TAC data that do not meet these criteria are not considered to be true drinking episodes and will be ignored. This macro evaluates days based on a 24-h “social day” from 6 AM to 6 AM.

### Magnetic resonance imaging

Participants completed a 1-h MRI acquisition on a Siemens Skyra 3 T MRI scanner (Siemens Medical Solutions, Erlangen, Germany) at the University of Miami. The 3D T1 weighted volumetric magnetization-prepared rapid gradient-echo sequence (MP-RAGE) consisted of 176 slices at slice thickness = 1 mm isotropic, FOV = 256×256, TR = 1.80s, and TE = 2.67 s. The resting-state functional MRI (rsfMRI) scan was administered for 8 min with eyes open consisting of 120 volumes and 48 interleaved slices at a slice thickness = 3.0 mm isotropic, FOV = 212×212, TR = 3.0 s, and TE = 30 ms.

### Functional MRI pre-processing

Functional MRI pre-processing was completed in accordance with past studies by our group (66). Specifically, functional images were preprocessed and analyzed using the MATLAB R2016b based functional connectivity toolbox “Conn toolbox” version 19c and SPM 12 (67, 68). We followed a pre-processing pipeline which included functional realignment and unwarping, functional centering of the image to (0, 0, 0) coordinates, slice-timing correction, structural centering to (0, 0, 0) coordinates, structural segmentation and normalization to MNI space, functional normalization to MNI space, and spatial smoothing with a kernel of 8 mm FWHM. During pre-processing, the Conn toolbox implements an anatomical, component-based, noise correction strategy (aCompCor) for spatial and temporal processing to remove physiological noise factors from the data (69). The implementation of aCompCor combined with the quantification of participant motion and the identification of outlier scans through the Artifact Rejection Toolbox (ART) allows for better interpretation of functional connectivity results (68–70). The ART was set to the 97th percentile setting with the mean global-signal deviation threshold set at *z* = ±3 and the participant-motion threshold set at 0.9 mm. As mentioned above, participants were removed if (1) global signal change was greater than 3SD from the group mean (one participant removed), (2) motion parameters exceeding 0.9 mm (two participants removed), and (3) number of invalid scans exceeding 20% of total scans (no participants removed). Applying linear regression and using a band-pass filter of 0.008–0.09 Hz, data were de-noised to exclude signal frequencies outside of the range of expected BOLD signals (such as low-frequency scanner drift), minimize participant motion, extract white matter and cerebral spinal fluid noise components, and control for within-participant realignment and scrubbing covariates.

### Within- and between-networks analysis of rsfMRI connectivity

For statistical analysis of rsfMRI connectivity, we used a publicly available network parcellation of the brain (71) that has been commonly used in the resting-state literature (72–79). From this atlas, we utilized five main networks to include: (1) the Cingulo-Opercular Network (consisting of the parietal operculum, temporal occipital cortex, frontal operculum, lateral prefrontal cortex); (2) Default Mode Network (prefrontal cortex, posterior cingulate cortex, parahippocampal cortex, and parietal and temporal cortices [corresponding to the angular gyrus and middle temporal gyrus, posterior division, respectively]), (3) the Dorsal Attention Network (posterior cortex [corresponding to the lateral occipital cortex, superior division], frontal eye fields, and precentral ventral cortex), (4) the Fronto-Parietal Control Network (parietal cortex [corresponding to the posterior division of the supramarginal gyrus], temporal cortex [corresponding to the posterior division of the middle temporal gyrus], dorsal prefrontal cortex, lateral prefrontal cortex, orbitofrontal cortex, ventral prefrontal cortex, medial posterior prefrontal cortex, precuneus, and the cingulate cortex), and (5) the Limbic Network (orbitofrontal cortex [corresponding to the frontal pole], temporal pole). The resting-state networks were registered to MNI152 space, and we defined the networks as regions of interests (ROIs) for ROI-ROI functional connectivity analyses. ROI-ROI analyses are Fisher z-transformed bivariate correlations between brain regions’ BOLD time-series that quantify associations in the activation at rest and serve as a proxy for functional connectivity. Using the Conn toolbox Results Explorer, within-network connectivity was calculated and compared with one or more variables of interest by computing the mean of the pairwise correlations between the specified ROIs that comprised each of the five higher-order functional networks. Between-networks connectivity comparisons were computed using the Conn Toolbox ROI-to-ROI approach, such that a seed’s BOLD time course signal is used as a reference for correlations followed via a search for any correlation with other ROIs’ positive and negative BOLD time course signal. Manual error correction settings were utilized such that all analyses were corrected for false discovery rate (FDR) at the connection level (p-FDR < 0.05) and at the cluster level with an MVPA omnibus test (p-FDR < 0.05).

### Statistical approach

PLWH and people without HIV were compared on demographic variables and alcohol use characteristics (both TLFB and TAC-AUC data) with *t*-test or Chi-square analysis, where appropriate. Variables where significant differences exist were inputted as covariates in any rsfMRI analyses comparing groups. Correlational analyses were completed to determine the relationship between all other measures were analyzed for normality and fit, with appropriate normalization applied where necessary.

### HIV status, within-network rsfMRI connectivity, and alcohol use: interactive associations

Statistical analyses were deployed to examine the effect of HIV status on connectivity at the pre- and post-CM periods as well as connectivity change as a function of self-reported and objective alcohol use (TAC-AUC) reduction. Connectivity values for each network at baseline, 30-day follow-up, and change from baseline to follow-up were centered by demeaning each participant’s connectivity value. An interaction term was then created between centered connectivity values and HIV status. Given the fact that baseline characteristics may influence later outcomes related to interventions, such as CM, HIV status, the centered connectivity values, and the interaction term were then entered into a linear regression equation predicting either TAC-AUC percent reduction or TLFB percent reduction. All assumptions for normality were met through examination of the P–P plots for potential heteroscedasticity. For interaction analyses, the direction for the overall model equation was computed for each group to confirm association slopes were opposing, as is often the case when an regression interaction is present.

## Results

The majority of participants were male (60%), identified as Black (74.3%) non-Hispanic (85.7%). Participants did not differ on demographic variables when comparing groups by HIV status (Table 1). PLWH did not differ from those living without HIV on any rsfMRI QC metrics related to motion, global signal change, or number of invalid scans.

**Table 1:** Demographics.

Variable	Total *N* = 35	PLWH *N* = 18	w/out HIV *N* = 17
Age (mean ± stdev)	57.2 ± 4.6	55.6 ± 3.9	57.9 ± 5.3
Current gender identity (%)			
Male	60.0%	55.6%	64.7%
Female	37.1%	38.9%	35.3%
Transgender	2.9%	5.6%	0%
Race (%)			
Caucasian	20.0%	22.2%	17.6%
Black	74.3%	77.8%	70.6%
American Indian/Alaska Native	0%	0%	0%
Native Hawaiian/Pacific Islander	0%	0%	0%
Asian	0%	0%	0%
Multi-racial	5.7%	0%	11.8%
Ethnicity (%)			
Hispanic	14.3%	11.1%	17.6%
Non-Hispanic	85.7%	88.9%	82.4%
Education level (%)			
Did not finish high school	28.6%	33.3%	23.5%
High school	34.3%	22.2%	47.1%
Greater than high school	37.1%	44.4%	29.4%
HIV disease duration (years)	–	18.82 (12.4)	–
Viral load (≥40 copies/mL)	–	0.24 (0.44)	–
CD4 Count (cells/μL)	–	600.28 (345.5)	–
Currently taking HIV antiretroviral medication	–	100%	–
Ever diagnosed with AIDS	0%	22.2%	0%
Years since first regular alcohol use^a^	37.74 (7.80)	36.44 (6.75)	39.12 (8.78)
TLFB-30 pre-CM (mean ± stdev)	8.44 (3.99)	8.53 (4.50)	8.35 (3.50)
TLFB-30 post-CM (mean ± stdev)	0.76 (1.62)	1.23 (2.14)	0.27 (0.44)
TLFB-30 percent reduction (mean ± stdev)	89.4% (20.1)	82.9% (26.2)*	96.3% (6.1)
TAC-AUC pre-CM (mean ± stdev)	27.50 (27.2)	24.51 (28.0)	30.67 (26.9)
TAC-AUC post-CM (mean ± stdev)	6.16 (13.2)	5.95 (14.1)	6.39 (12.6)
TAC-AUC percent reduction (mean ± stdev)	60.5% (93.3)	45.5% (114.3)	75.5% (66.7)

*PLWH and people without HIV differ (*p* < 0.05); ^a^Defined as using alcohol at least once per week.

TLFB, Timeline Follow-Back 30; TAC-AUC, transdermal alcohol content (TAC) area under the curve; TLFB pre-CM, the reported daily average number of drinks over the 30 days prior to contingency management; TLFB post-CM, the reported daily average number of drinks over the 30 days of contingency management; TAC-AUC pre-CM, average daily AUC over the 7 days prior to contingency management; TAC-AUC post-CM, average daily AUC over the final 7 days of the contingency management period; Percent Reduction = [(Baseline – Follow-up) / Baseline]*100.

### Pre- and post-contingency management alcohol consumption

On the pre-CM TLFB-30, participants reported a mean of 253.3 (SD = 119.7) standard drinks consumed, or 8.44 drinks per day (SD = 3.99), on average. During the 7 days leading up to the intervention, the average daily TAC-AUC was 27.5 h · g/dl (SD = 27.2). Over the 30 days of intervention, participants self-reported an average of 22.8 (SD = 48.6) drinks on the TLFB-30, or 0.76 drinks per day (SD = 1.62). During the final 7 days of the 30-day intervention, the average daily TAC-AUC was 6.16 h · g/dl (SD = 13.2). In terms of percent reduction in alcohol consumption, results indicate an 89.4% reduction in average daily self-reported alcohol use on the TLFB-30 and a 60.5% reduction in average daily objective alcohol use on TAC-AUC (Table 1).

### Baseline functional connectivity

**Figure 1:**
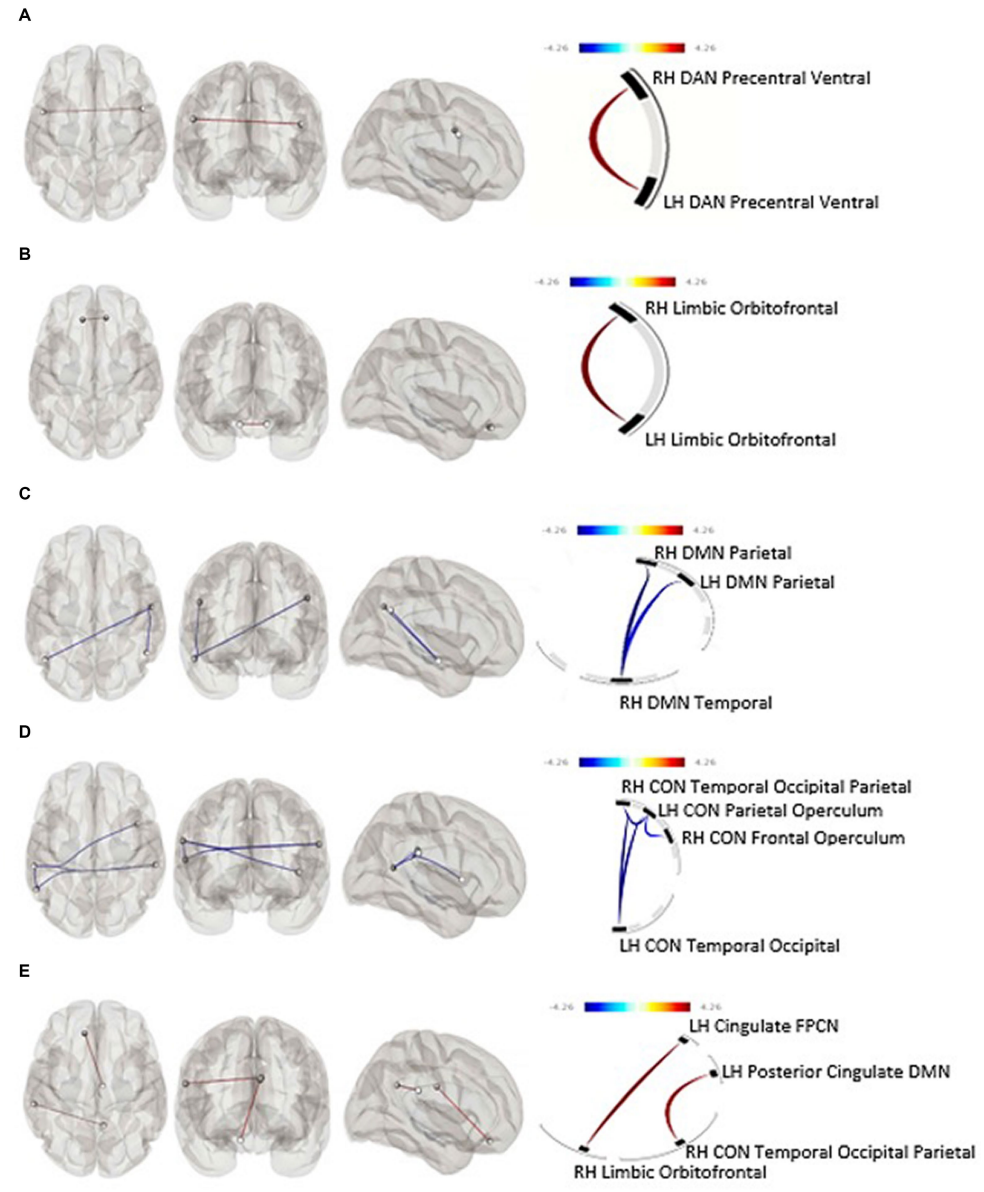
**(A)** Within-networks baseline rsfMRI and subjective pre-intervention drinking (p-FDR = 0.0001), **(B)** within-networks baseline rsfMRI and predicted 30-day subjective percent drinking reduction (p-FDR = 0.046), **(C)** within-networks 30-day rsfMRI and predicted 30-day subjective percent drinking reduction, **(D)** within-networks baseline rsfMRI and objective drinking reduction (p-FDR = 0.039), **(E)** between-networks pre-post rsfMRI change and objective drinking reduction. Blue line, positive association between connectivity and drinking reduction; Red line, negative association between connectivity and drinking reduction; CON, Cingulo-Opercular Network; DAN, Dorsal Attention Network; DMN, Default Mode Network; FPCN, Fronto-Parietal Control Network; LH, Left Hemisphere; RH, Right Hemisphere.

Average TAC-AUC for objective drinking over the 7 days prior to the intervention was not significantly associated with functional connectivity at baseline after correction for false discovery rate (FDR). Baseline functional connectivity within two bilateral nodes of the DAN was positively associated with higher self-reported (TLFB) pre-CM drinking over the seven days immediately before the MRI session (F[1,33] = 24.96, p-unc = 0.000019, p-FDR = 0.00012); an association occurring in the opposite direction of our initial hypothesis (Figure 1A).

Baseline functional connectivity between multiple nodes within the CON was negatively associated with TAC-AUC reduction after CM (Cluster 1: F[2,29] = 5.86, p-unc = 0.007303, p-FDR = 0.038744; Cluster 2: F[2,29] = 5.77, p-unc = 0.0077, p-FDR = 0.0387) (Figure 1D). Baseline functional connectivity within the bilateral orbitofrontal nodes of the limbic network was positively associated with subjective drinking reduction after 30 days of intervention (F[1,33] = 6.53, p-unc = 0.0154, p-FDR = 0.0462) (Figure 1B).

### Functional connectivity at the post-CM period: effect of drinking reduction

Neither within- nor between-networks functional connectivity at the 30-day follow-up was associated with drinking reduction as determined by percent reduction of TAC-AUC. Within-network DMN functional connectivity at the 30-day follow-up was significantly negatively associated with self-reported drinking reduction on the TLFB (F[2,32] = 7.68, p-unc = 0.0019, p-FDR = 0.0113) (Figure 1C).

### Post-CM change in functional connectivity

After 30-days of attempted abstinence from heavy drinking, between-networks functional connectivity significantly increased as a function of objective reduction in alcohol use as determined by TAC-AUC. Specifically, a connectivity increase was observed between the limbic and frontoparietal control network (t[30] = 2.54, *p* = 0.008) driven by the cingulate region of the fronto-parietal control network and the orbitofrontal cortex of the limbic network (F[2,25] = 8.27, p-unc = 0.0017, p-FDR = 0.0315) (Figure 1E). There were no within-network associations observed between functional connectivity change and objective drinking reduction via TAC-AUC. Similarly, no within- or between-network changes in functional connectivity were observed as a function of self-reported drinking reduction on the TLFB.

### HIV associations with rsfMRI connectivity

**Figure 2:**
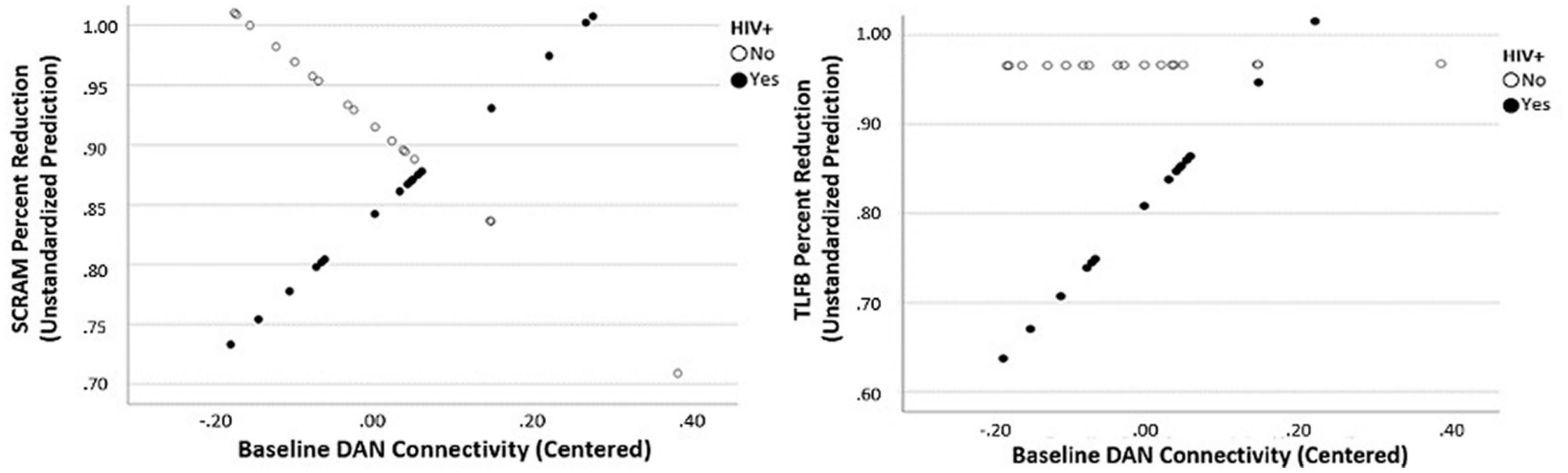
Interactive effect of baseline Dorsal Attention Network connectivity predicting percent reduction in objective drinking (SCRAM) and subjective drinking (TLFB) as a function of HIV status.

As seen in the lower half of Table 1, PLWH demonstrated a statistically lower percent reduction in self-reported drinking compared to people without HIV (t[33] = −2.12, *p* = 0.047), thus necessitating a comparison of these groups when examining the effect of intervention on resting-state connectivity. Results revealed a significant main effect of HIV diagnosis and DAN connectivity at baseline for self-reported percent alcohol reduction (t[34] = 20.5; *p* < 0.001) as well as a significant interactive effect of HIV status and baseline DAN connectivity (t[34] = 2.11; *p* = 0.043). There was also a significant main effect of HIV status and DAN connectivity at baseline for objective percent drinking reduction on SCRAM (t[31] = 20.0; *p* < 0.001) as well as a significant interactive effect of HIV status and baseline DAN connectivity (t[31] = 2.63; *p* = 0.014) (Figure 2). There were no main effects of HIV status on objective or self-reported percent drinking reduction for any of the five networks at the 30-day follow-up. Similarly, there were no main effects of HIV status on objective or self-reported percent drinking reduction for change in connectivity within the five networks examined.

## Discussion

Although recent advances in neuroimaging techniques have provided insights into functional changes associated with heavy drinking, longitudinal studies assessing functional mechanisms of drinking reduction are rare, especially in PLWH. Following short-term abstinence from alcohol, the current literature indicates that individuals experience greater activity in limbic-striatal regions and lower activity in the medial frontal and cingulated regions, suggestive of difficulties with emotional regulation, self-control, and executive functioning (28, 35). As such, the purpose of the present study was to determine the ability of baseline resting-state functional MRI (rsfMRI) connectivity to predict later reduction in heavy alcohol use as well as how rsfMRI connectivity is altered after 30 days of attempted abstinence from heavy alcohol use. The present study adds to the neuroimaging literature on AUD by identifying changes in resting state functional connectivity associated with drinking reduction, rather than total abstinence, following a contingency management intervention. Furthermore, given that no study to date has examined the association of wearable biosensor detected alcohol use and brain biomarkers, we compared measures of objective (biosensor-based) and subjective (self-report) drinking reduction to neurobiological changes in individuals living with and without HIV.

Results of the present analysis indicated increased between-networks connectivity between the limbic and fronto-parietal control networks in those who demonstrated higher objective abstinence from alcohol. Given the unique nature of the present study, extant literature is limited when investigating between-networks change in rsfMRI connectivity after alcohol reduction. However, this finding suggests a potential improvement in functional network coherence given that previous rsfMRI studies in clinical populations have demonstrated widespread decreases in intranetwork and internetwork correlations, such as in normal aging (80) as well as with increasing severity of Alzheimer’s Disease (77, 78). Alternatively, it is possible that increased between-networks connectivity may indicate a “bleeding together” of the typically disparate co-activation patterns due to disruption of physiological and/or neurological homeostasis associated with prolonged alcohol use. However, to confirm that this increase in between-networks connectivity was not the result of decreased network segregation, we additionally completed a segregation analysis following previously described methods (81, 82). These post-hoc findings suggested an overall net increase in between-networks connectivity, indicating increased between-networks segregation from pre-to-post-intervention. These results suggest that these networks maintained unique network connectivity patterns following alcohol reduction in our sample, providing further confidence that the changes in resting-state synchronicity following alcohol reduction indicates improved functional connectivity.

The second major finding of our analysis was lower resting-state functional connectivity of the salience network at baseline was associated with greater post-intervention drinking reduction in individuals who exhibit heavy alcohol consumption. The salience network refers to the co-activation of brain regions, such as the anterior cingulate and ventral anterior insular cortices, as well as the amygdala, hypothalamus, ventral striatum, thalamus, and specific brainstem nuclei with domain-specific functions separating the most relevant internal and extra-personal stimuli to guide goal-directed behavior (17). According to the neuro-circuitry model of addiction, these two brain regions appear to influence the dynamics between large-scale brain networks enhancing salience for alcohol-related cues while inhibiting the response to other stimuli (83, 84). Therefore, our results suggest that individuals who engage in heavy alcohol use and have lower Salience network resting state functional connectivity at baseline may be less susceptible to the environmental (e.g., alcohol advertisements, billboards, etc.) and social cues (e.g., social gatherings, peer pressure, etc.) that tend to make attempts at alcohol abstinence unsuccessful. In other words, the appetitive synchrony in the salience network associated with long-term alcohol use may render individuals with AUD more vulnerable to alcohol craving and repeated consumption, but individuals with lower resting state functional connectivity in these areas at rest may be better able to engage in drinking reduction approaches.

This study also revealed some important findings in regard to differences between self-reported alcohol use and objective alcohol use in how they relate to baseline rsfMRI connectivity as well as change in connectivity after alcohol reduction. Self-reported alcohol use but not objective alcohol use metrics in the pre-CM period was related to baseline DAN connectivity. As such, it is possible that increased baseline engagement of this network may be related to the cognitive ability for self-apprasial and self-reporting alcohol use, but not for actual alcohol consumption itself. Such an interpretation would reveal why objective alcohol use is not related to baseline DAN connectivity and why DAN connectivity at baseline predicts alcohol reduction after intervention without a post-CM change in DAN functional connectivity. We also found a positive association between limbic connectivity and subjective report of alcohol use reduction after CM (85). Previous studies in other clinical populations, such as those suffering from major depressive disorder (MDD) have found depression symptoms to be related to limbic network connectivity and that increased connectivity in this network is observed after antidepressant treatment. As such, it is possible that mood alterations resulting from decreased alcohol use are driving associations of self-reported drinking reduction and limibic connectivity (86). Further, one question this investigation elicits is in regard to the lack of rsfMRI connectivity associations with drinking reduction at the follow-up period despite associations with *change* in rsfMRI connectivity. It is possible that change in drinking, as a metric, is more readily associated with change in rsfMRI connectivity, but that other health or mood factors at follow-up, such as improved ART medication adherence or depressive symptom reporting, are more strongly linked to an individual’s present rsfMRI connectivity patterns after drinking reduction. Such a study would provide increased clinical utility of rsfMRI connectivity in relation to drinking reduction and its effect on clinically-applicable and measurable changes after intervention. As such, future investigations should examine medication adherence, depression symptom reporting, and other health-related factors in relation to enduring rsfMRI connectivity patterns after a short period of drinking reduction.

Finally, results revealed a significant main effect of HIV diagnosis and DAN connectivity at baseline for self-reported percent alcohol reduction. Additionally, there was a significant interactive effect of HIV status and baseline DAN connectivity such that higher DAN connectivity at baseline was predictive of greater drinking reduction in PLWH. The DAN is thought to underlie selective visual attention (87, 88), which is particularly important for goal-directed behavior. Previous rsfMRI research has demonstrated abnormal functional connectivity in the DAN in patients with alcohol-use disorder (89). Therefore, our findings may suggest a disruption of DAN connectivity in PLWH who use alcohol, making it more difficult for individuals living with HIV to maintain abstinence. This finding also ties into the previously described finding of decreased salience network connectivity predicting greater post-intervention alcohol reduction. As the DAN is engaged during externally directed attention, salient environmental cues may be particularly important for reinforcing alcohol consumption in PLWH with heavy alcohol use histories. Important for the present study, our data suggest that PLWH with disrupted DAN connectivity may not benefit as greatly from CM for alcohol reduction as those with higher levels of within-network DAN connectivity at baseline.

We acknowledge some limitations in the current study. First, while this study included a longitudinal design with two data points per participant, the sample size was relatively small and thus limiting power and generalizability. Further, participants in the present study were moderate-to-heavy drinkers but were not dependent on alcohol. Therefore, resting state synchrony should be examined in a larger sample comprising adults with greater severity of alcohol dependence. Additionally, this study examined a set of statistical inferences simultaneously, lending to an issue of multiple comparisons. However, when all networks were evaluated in a single model liberally correcting for multiplicity, increased between-networks connectivity in those with higher abstinence remained significant, suggesting that this is a robust finding despite a small sample size. Relatedly, some HIV-related factors that may have influenced the findings were not considered due to the limited sample size, and future analyses should examine the impact of HIV duration, viral load, AIDS, antiretroviral therapy (ART), duration on ART, CD4 nadir, and CD4 t-cell count. Third, this study was not a randomized clinical trial, meaning participants were not randomized to intervention or control groups. Therefore, conclusions cannot be drawn regarding the efficacy of the contingency management intervention as all participants had the opportunity to receive financial incentives to reduce drinking. Future research should examine the use of non-monetary incentives for drinking reduction (e.g., vouchers, prizes, etc.). Similarly, the alcohol reduction and CM effects on rs-fMRI networks cannot be differentiated in this study. Despite these weaknesses, this is the first study to our knowledge that examined resting state synchrony pre- and post-drinking reduction in a sample of individuals with and without HIV.

## Conclusion

Overall, the present study sought to determine the effects of 30-days drinking cessation/reduction on resting state functional connectivity in people with and without HIV. We found that lower resting-state functional connectivity of the Salience network significantly predicted stronger drinking reduction, and greater drinking reduction following CM was associated with increased between-networks connectivity. Consistent with previous research, our findings also demonstrated a disruption of DAN connectivity in PLWH who use alcohol, making it more difficult for individuals living with HIV and who had lower DAN connectivity to maintain abstinence with CM; this finding was corroborated by results indicating that PLWH with lower DAN connectivity were less successful at drinking reduction following CM compared to those without HIV. These findings not only suggest a potential biomarker for reduced susceptibility to the environmental and social cues that often make alcohol use reduction attempts unsuccessful, but also indicate that individuals experience benefits in brain connectivity associated with reduced drinking. Therefore, FC alterations associated with chronic alcohol use may be reversible, and may serve as clinical biomarkers of change in drinking behaviors for future studies which do not employ wearable biosensor monitoring devices.

## Data availability statement

The raw data supporting the conclusions of this article will be made available by the authors by request, without undue reservation.

## Ethics statement

The studies involving human participants were reviewed and approved by the University of Florida Institutional Review Board and University of Miami Institutional Review Board. The patients/participants provided their written informed consent to participate in this study.

## Author contributions

JG contributed to conception and design of the study and performed the statistical analysis. JG and JD wrote the manuscript. ZZ organized the database. RAC and RLC acquired funding for the study. VG and TS supervised the data collection. All authors contributed to the manuscript revision, read, and approved the submitted version.

## Funding

This project was supported by NIH grants T32-DA017629 and U01-AA020797-10.

## Conflict of interest

The authors declare that the research was conducted in the absence of any commercial or financial relationships that could be construed as a potential conflict of interest.

## Publisher’s note

All claims expressed in this article are solely those of the authors and do not necessarily represent those of their affiliated organizations, or those of the publisher, the editors and the reviewers. Any product that may be evaluated in this article, or claim that may be made by its manufacturer, is not guaranteed or endorsed by the publisher.
